# Time-related survival prediction in molecular subtypes of breast cancer using time-to-event deep-learning-based models

**DOI:** 10.3389/fonc.2023.1147604

**Published:** 2023-06-05

**Authors:** Saba Zarean Shahraki, Mehdi Azizmohammad Looha, Pooya Mohammadi kazaj, Mehrad Aria, Atieh Akbari, Hassan Emami, Farkhondeh Asadi, Mohammad Esmaeil Akbari

**Affiliations:** ^1^ Department of Health Information Technology and Management, School of Allied Medical Sciences, Shahid Beheshti University of Medical Sciences, Tehran, Iran; ^2^ Basic and Molecular Epidemiology of Gastrointestinal Disorders Research Center, Research Institute for Gastroenterology and Liver Diseases, Shahid Beheshti University of Medical Sciences, Tehran, Iran; ^3^ Geographic Information Systems Department, Faculty of Geodesy and Geomatics Engineering, K. N. Toosi University of Technology, Tehran, Iran; ^4^ Faculty of Information Technology and Computer Engineering, Azarbaijan Shahid Madani University, Tehran, Iran; ^5^ Cancer Research Center, Shahid Beheshti University of Medical Sciences, Tehran, Iran

**Keywords:** breast cancer survival prediction, breast cancer molecular subtypes, survival prediction models, survival analysis, time-to-event machine learning models, deep learning survival models, feature importance, AI application in breast cancer

## Abstract

**Background:**

Breast cancer (BC) survival prediction can be a helpful tool for identifying important factors selecting the effective treatment reducing mortality rates. This study aims to predict the time-related survival probability of BC patients in different molecular subtypes over 30 years of follow-up.

**Materials and methods:**

This study retrospectively analyzed 3580 patients diagnosed with invasive breast cancer (BC) from 1991 to 2021 in the Cancer Research Center of Shahid Beheshti University of Medical Science. The dataset contained 18 predictor variables and two dependent variables, which referred to the survival status of patients and the time patients survived from diagnosis. Feature importance was performed using the random forest algorithm to identify significant prognostic factors. Time-to-event deep-learning-based models, including Nnet-survival, DeepHit, DeepSurve, NMLTR and Cox-time, were developed using a grid search approach with all variables initially and then with only the most important variables selected from feature importance. The performance metrics used to determine the best-performing model were C-index and IBS. Additionally, the dataset was clustered based on molecular receptor status (i.e., luminal A, luminal B, HER2-enriched, and triple-negative), and the best-performing prediction model was used to estimate survival probability for each molecular subtype.

**Results:**

The random forest method identified tumor state, age at diagnosis, and lymph node status as the best subset of variables for predicting breast cancer (BC) survival probabilities. All models yielded very close performance, with Nnet-survival (C-index=0.77, IBS=0.13) slightly higher using all 18 variables or the three most important variables. The results showed that the Luminal A had the highest predicted BC survival probabilities, while triple-negative and HER2-enriched had the lowest predicted survival probabilities over time. Additionally, the luminal B subtype followed a similar trend as luminal A for the first five years, after which the predicted survival probability decreased steadily in 10- and 15-year intervals.

**Conclusion:**

This study provides valuable insight into the survival probability of patients based on their molecular receptor status, particularly for HER2-positive patients. This information can be used by healthcare providers to make informed decisions regarding the appropriateness of medical interventions for high-risk patients. Future clinical trials should further explore the response of different molecular subtypes to treatment in order to optimize the efficacy of breast cancer treatments.

## 1 Introduction

Breast cancer (BC) is the most prevalent cancer in women worldwide, with 2.3 million new BC cases and 685,000 deaths in 2020 (1). Accurate survival prediction of BC can help healthcare providers to better understand patients’ prognosis and prevent unnecessary medical interventions (2).

Traditionally, BC is defined based on different clinical and histological characteristics, including tumor grade, stage of tumor, and hormone receptor status (3, 4). Immunohistochemistry (IHC) receptors, i.e., estrogen receptor (ER), progesterone receptor (PR), and human epidermal growth factor receptor 2 (HER2), are used to categorize BC tumors into four subtypes: luminal A (ER-positive/or PR-positive/HER2-negative), luminal B (ER-positive/or PR-positive/HER-positive), triple negative (ER-negative/PR-negative/HER-negative), and HER2-enriched (ER-negative/PR-negative/HER2-positive) (5). Recent research has used IHC subtypes to characterize BC survival, and has assessed BC survival in both long- and short-term examinations, as each IHC subtype was found to respond differently to adjuvant therapies. Characterizing these markers is likely critical to improving BC patients’ survival and treatments in clinical practice (6, 7).

The Cox regression model has been widely used to analyze time-to-event data for the survival of BC molecular subtypes (8–10). As a result of the rapid development in machine learning and, in particularly neural networks, and its applications in healthcare and medical purposes (11–13), a number of new methods for time-to-event predictions have been developed in recent years (14). To the best of our knowledge DeepHit (15), DeepSurv (16), Neural multi-task logistic regression (NMTLR) (17), Cox-Time (18), and Nnet-survival (19) models have shown high performances in predicting survival probabilities using various clinical datasets.

This study aimed to develop time-to-event survival prediction models using a large-scale Iranian institutional BC dataset to identify the best-performing model for BC patients. The objective of this study was to use this model to predict survival probabilities of four molecular subtypes over a 30-year follow-up period, in order to evaluate BC survival outcomes in both short- and long-term intervals. Additionally, feature importance was utilized to obtain an optimized model.

## 2 Materials and methods

### 2.1 Study design

This retrospective cohort study included 5,362 patients diagnosed with invasive BC between 1991 and 2021 at the Cancer Research Center (CRC) affiliated with Shahid Beheshti University of Medical Science (SBMU) in Tehran. Data from patients who underwent surgery followed by adjuvant treatments were extracted from the electronic health records registered in the Breast Cancer Registry System at the CRC. The study was approved by the CRC and the Ethics Committee of SBMU (IR.SBMU.RETECH.REC.1395.750).

### 2.2 Study population

For each patient, demographic and clinical factors related to BC were collected. Demographic factors included age at diagnosis (Mean ± SD = 48.83 ± 11.59), education level (higher education/high school diploma/middle school/elementary school/illiterate), marital status (married/not married), gravidity (the number of times a patient has been pregnant), breastfeeding duration (0/less than 2 years/between 2-4 years/between 4-6 years/more than 6 years), abortion history (yes/no), and BC family history (none/1st degree/2nd degree). Clinical and pathological factors included morphology (invasive ductal carcinoma/invasive lobular carcinoma), lymph vascular invasion (yes/no), tumor size (T1/T2/T3), lymph node status (N0/N1/N2/N3), tumor histological grade (low/intermediate/high), tumor stage (I/II/III/IV) and molecular subtypes (subgroup 1 - ER+ or PR+/HER2+, subgroup 2 - ER+ or PR+/HER2-, subgroup 3 - ER- and PR-/HER2+, and subgroup 4 - ER- and PR-/HER2-), type of surgery performed (breast conserving surgery/modified radical mastectomy), chemotherapy received (none/adjuvant/nonadjuvanted), radiotherapy administered (none/external/intraoperative) and hormonotherapy prescribed (yes/no) were also used in the analysis. In total, 18 time-independent variables were collected for each patient.

The pathological stage of BC was obtained according to the criteria of the 7th edition of the American Cancer Committee (20). All ER, PR, and HER2 results were identified by the IHC testing. HER2 amplification for patients with equivocal IHC results (2+ grade) was assessed by Fluorescence in Situ Hybridization (FISH) or Chromogenic in Situ Hybridization (CISH) analysis (21, 22). Cases were classified as HER2 negative if their FISH or CISH test were negative or they had an IHC score of 0 or 1+ and were classified as HER2 positive with positive FISH or CISH test or with an IHC score of 3+. For each patient, 1) time-independent variables, 2) the time between the patient’s first diagnosis and the time of the patient’s death or last visit, and 3) a label indicating the survival status of the patient (censored or dead) were calculated.

### 2.3 Data quality

Prior to analysis, the dataset was assessed for quality issues. These included multiple recorded data for some patients, undetermined IHC status, and outdated survival status. Additionally, some breast cancer diagnoses were incorrectly recorded, patient ages and dates of birth were mismatched, and the small number of male patients could lead to potential biases in the results. To address these issues, the data was cleaned up in several steps. First, the accuracy of patient birthdays and cancer diagnosis age were verified and any incorrect information was modified or removed. Cases with missing IHC status, unknown pathology, non-invasive and non-popular BC, and patients who developed second primary BC were excluded. Duplicate cases were identified by checking their first name and surname, sex, and father’s name. Patients with exactly matched records were assigned to duplicate records and were removed automatically. The status of patients’ survival was updated by contacting their families. Patients who could not be reached, those who died through non-cancer causes, and those who were alive were considered censored, while those who died due to BC were considered dead.

### 2.4 Feature importance

To determine the most important prognostic variables that affected BC survival time, the Breiman-Cutler permutation method was used with the random forest algorithm (23–25). The dataset was divided into a training set of 80% (n=2864) and a test set of 20% (n=716). The training dataset was analyzed using the random forest algorithm, and parameters such as mtry (number of variables to be split at each node) and node size (minimum size of the terminal node) were adjusted based on analyzing out-of-bag errors. The variables were then selected using the tuned random forest with test data based on the mean decrease in accuracy. Higher values of mean decrease in accuracy indicate greater importance of a variable in predicting survival time. If a variable is associated with the survival probability, this permutation will lead to a decrease in prediction accuracy (26). All analyses were conducted using R (4.2.1) and SPSS (version 26). P-values less than 0.05 were considered statistically significant.

### 2.5 Model training and performance

Time-to-event models (i.e., DeepHit, NMTLR, Nnet-survival, and DeepSurve and Cox-Time) available in the Pycox^1^ package were used to analyze the survival data. All models were constructed using all variables and the variables obtained from the feature importance section.

**DeepHit:** this model utilizes a neural network to estimate the joint distribution of survival time and event, while accounting for the inherent right-censored nature of survival data. The model utilizes a fully parametric approach to predict failure times over a discrete set of fixed size, incorporating both survival times and relative risks in its loss function.

**Neural Multi-Task Logistic Regression (N-MTLR):** this model is an extension of the Linear Multi-Task Logistic Regression (MTLR) technique that utilizes a deep learning architecture to address the linearity problem in modeling nonlinear dependencies in the dataset. The MTLR model is used to jointly model binary labels representing event indicators at different time intervals, allowing for the assessment of the probability of an event occurring within each interval (27).

**Nnet-survival:** this model is a discrete-time survival model for neural networks that incorporates non-proportional hazards and can be trained with mini-batch gradient descent. The model is theoretically justified as it uses the likelihood function as the loss function, enabling fast training and avoiding local minimums of the loss function.

**DeepSurv:** this model is an integration of the Cox proportional hazards model with neural networks that can learn complex relationships between an individual’s covariates and the effect of a treatment. The model utilizes a core hierarchical structure composed of fully connected feed-forward neural networks with a single output node and uses the negative log partial likelihood function to assess patients’ survival hazards.

**Cox-Time:** this model is an extension of the Cox proportional hazards model that uses neural networks to parameterize the relative risk function and employs a batch-computable loss function, enabling scalability to large datasets. This model incorporates time as an additional input feature to capture its interactions with other input features, allowing for the modeling of complex relationships between covariates and event times, as well as interactions between covariates and time, without being limited by the proportionality assumption.

#### 2.5.1 Data preprocessing

Entity embedding were implemented to one-encode the categorical variables by using the half size of the number of categories (28). The entire dataset was randomly divided into 80 and 20 percent exclusive sets for training and testing the models respectively. This was achieved using the train_test_split method from the scikit-learn^2^ module, with the stratified argument assigned to molecular subtypes to ensure equal proportions were retained for each breast cancer subtype.

#### 2.5.2 Model design and hyperparameter tuning

With a focus on achieving the best-performing model, we tried to develop the neural network for each model. All models used a standard multilayer perceptron neural network as the model architecture to learn relationships between linear and nonlinear data. In order to develop discrete-time models, it was necessary to categorize survival time into optimal intervals (17, 18). We employed previous research methods (19) that were used to determine the optimal width of time intervals for discrete-time models (DeepHit, MLTR, Nnet-survival). Accordingly, the value of 10, which equated in approximately 36 months, was selected as the width of time intervals.

In order to determine the optimal hyperparameters for the neural network, a grid search was conducted using the Scikit-Learn library. The resulting values are presented in **Table 1**. Each training setup was trained with a batch size of 256 and utilized the Adam algorithm as an optimizer function due to its efficient runtime. To compensate for the small number of samples and having general models, 5-fold cross-validation was used for training each setup derived from the grid search (29). Specifically, four folds were used for training and one fold was reserved for testing in each iteration, resulting in five fully trained models for every combination of grid search and training dataset. Early stopping with a patience number of 10 was implemented to expedite the training process; if a model’s loss score did not improve after 10 consecutive epochs, the training process ceased and the best evaluation score was recorded for that fold. Additionally, in each training setup, 10 percent of the four folds were randomly selected and utilized as a validation set. Altogether, 450 distinct trainings consisting of 5-fold cross-validation for 90 grid search combinations were performed. The neural network structures were implemented in Python using the Pytorch library (Python 3.6, Pytorch 1.12.1).

**Table 1:** Grid search hyperparameters.

Hyperparameter	param-1	param-2	param-3	param-4	param-5	param-6
learning-rate	**0.1**	0.01	0.001	–	–	–
layers & nodes	[32, 32]	[32, 64]	[32, 64, 128]	**[32, 64, 128, 256]**	[32, 64, 128, 256, 512]	–
dropout	0.0	0.1	0.2	**0.3**	0.4	0.5

The bold values represent the tuned hyperparameters resulted from the grid search approach.

The symbol “–” means that the corresponding value or field is empty or has no data.

The output of each model was a 10-dimensional vector, where each element represented the predicted survival probability over each time interval (36 months). To visualize the Kaplan-Meier curves and compare the survival probabilities of each subtype across different time intervals, the 5-, 10- and 15-year mean survival probabilities for all patients in each specific time interval were calculated.

#### 2.5.3 Performance evaluation

The performance of the five models was evaluated using the concordance index (C-index) (30) as an evaluation metric. The C-index is a correlation coefficient that measures the degree of agreement between predicted survival risks and observed survival times. A C-index value of 0.5 indicates random prediction, while a value of 1.0 indicates excellent prediction. Additionally, the Integrated Brier score (31) (IBS) was used to assess the models’ calibrations by indicating the mean square difference between observed patient status and predicted survival probability, with scores ranging from 0 to 1 and lower scores indicating better performance. A Brier score below 0.25 is considered useful in practice. The model with the highest C-index and IBS scores among the five training folds of the grid search setups was selected as the best-performing model for predicting survival probability.

## 3 Results

### 3.1 Baseline characteristics and overall survival rate

**Figure 1:**
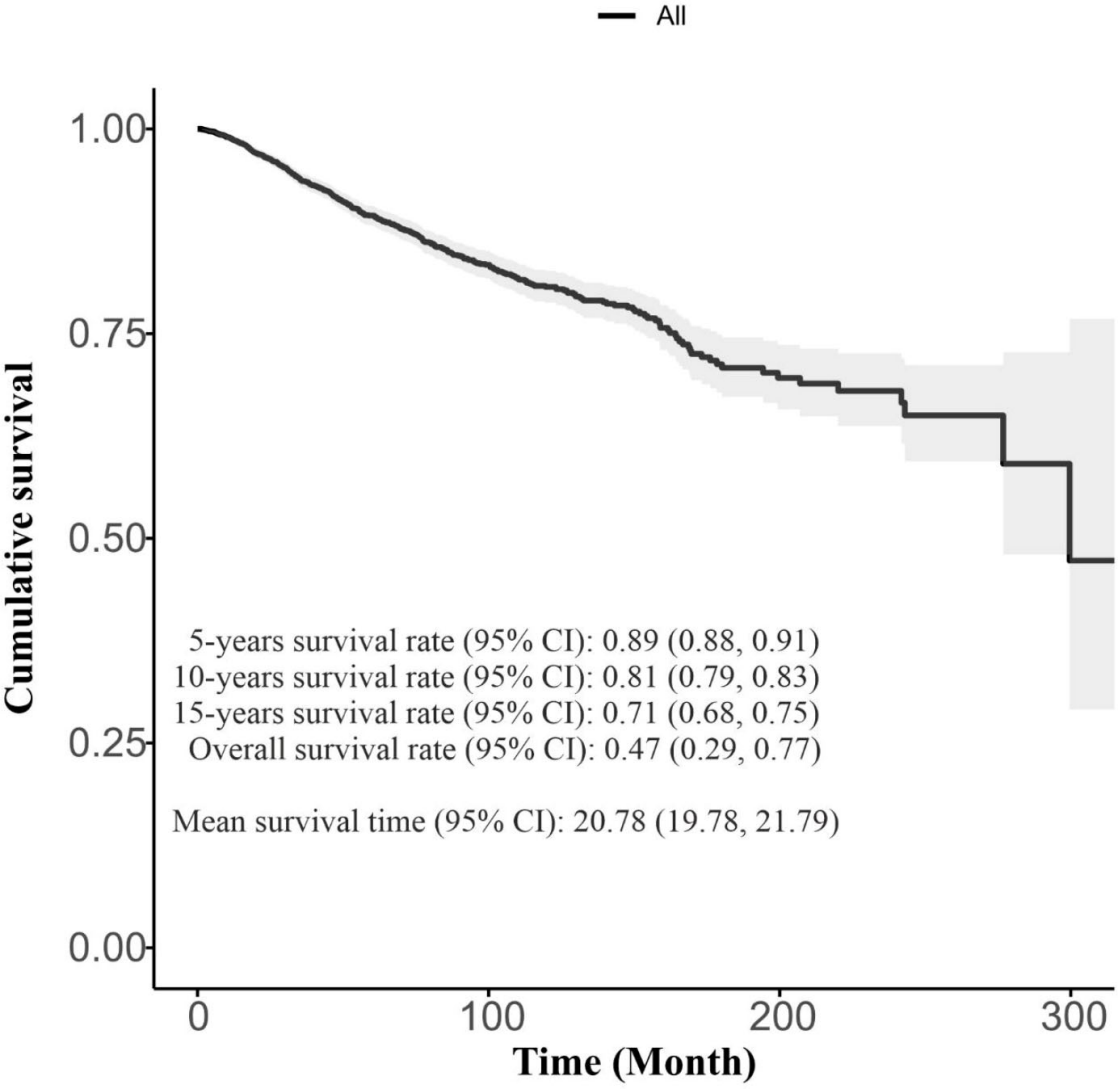
The Overall Kaplan-Meier survival Curve with summarization of survival times.

A total of 3580 women were included in the study, with a mean ( ± SD) age of 48.83 ± 11.59 years. 434 cases (12.1%) died during the study period due to the BC, while 3146 cases were censored. **Table 2** presents the demographic and clinical characteristics of BC patients. The overall survival rate (95% confidence interval [CI]) was 0.47 (0.29, 0.77) with a 5-years survival rate (95% CI) of 0.89 (0.88, 0.91). The mean survival time (95% CI) was 20.78 (19.78, 21.79) years, as illustrated in **Figure 1**.

**Table 2:** Description of variables in the dataset.

Variable	Levels	Total (n=3580)	Status		P-value
			Censored (n=3146)	Deceased (n=434)	
Age at diagnosis (years)	Mean ± SD	48.83 ± 11.59	48.59 ± 11.30	50.51 ± 13.38	0.005
Education	Higher education	1293 (36.12%)	1188 (37.76%)	105 (24.19%)	<0.001
	Highschool diploma	1327 (37.07%)	1188 (37.76%)	139 (32.03%)	
	Middle school	402 (11.23%)	333 (10.58%)	69 (15.90%)	
	Elementary school	353 (9.86%)	288 (9.15%)	65 (14.98%)	
	Illiterate	205 (5.73%)	149 (4.74%)	56 (12.90%)	
Marital status	Not married	539 (15.06%)	471 (14.97%)	68 (15.67%)	0.704
	Married	3041 (84.94%)	2675 (85.03%)	366 (84.33%)	
Gravidity	0	426 (11.90%)	384 (12.21%)	42 (9.68%)	<0.001
	1-2	1320 (36.87%)	1182 (37.57%)	138 (31.80%)	
	3-4	1260 (35.20%)	1103 (35.06%)	157 (36.18%)	
	More than 4	574 (16.03%)	477 (15.16%)	97 (22.35%)	
Abortion	No	2466 (68.88%)	2163 (68.75%)	303 (69.82%)	0.654
	Yes	1114 (31.12%)	983 (31.25%)	131 (30.18%)	
Breastfeeding duration	0	585 (16.34%)	529 (16.82%)	56 (12.90%)	0.001
	Less than 2 years	1308 (36.54%)	1130 (35.92%)	178 (41.01%)	
	2-4 years	1396 (38.99%)	1246 (39.61%)	150 (34.56%)	
	4-6 years	291 (8.13%)	241 (7.66%)	50 (11.52%)	
	More than 6 years	0 (0.00%)	0 (0.00%)	0 (0.00%)	
Family history	None	2601 (72.65%)	2283 (72.57%)	318 (73.27%)	0.382
	1st degree	514 (14.36%)	460 (14.62%)	54 (12.44%)	
	2nd degree	465 (12.99%)	403 (12.81%)	62 (14.29%)	
Tumor size	T1	1077 (30.08%)	1006 (31.98%)	71 (16.36%)	<0.001
	T2	1640 (45.81%)	1447 (45.99%)	193 (44.47%)	
	T3	863 (24.11%)	693 (22.03%)	170 (39.17%)	
Lymph node status	N0	1803 (50.36%)	1678 (53.34%)	125 (28.80%)	<0.001
	N1	1006 (28.10%)	901 (28.64%)	105 (24.19%)	
	N2	528 (14.75%)	406 (12.91%)	122 (28.11%)	
	N3	243 (6.79%)	161 (5.12%)	82 (18.89%)	
Tumor stage	I	725 (20.25%)	700 (22.25%)	25 (5.76%)	<0.001
	II	1528 (42.68%)	1416 (45.01%)	112 (25.81%)	
	III	1160 (32.40%)	944 (30.01%)	216 (49.77%)	
	IV	167 (4.66%)	86 (2.73%)	81 (18.66%)	
Tumor grade	Low	362 (10.11%)	333 (10.58%)	29 (6.68%)	0.016
	Intermediate	2044 (57.09%)	1799 (57.18%)	245 (56.45%)	
	High	1174 (32.79%)	1014 (32.23%)	160 (36.87%)	
Molecular Subtypes	Subgroup 1: ER + or PR +/HER2 +	503 (14.05%)	443 (14.08%)	60 (13.82%)	<0.001
	Subgroup 2: ER + or PR +/HER2 -	2175 (60.75%)	1961 (62.33%)	214 (49.31%)	
	Subgroup 3: ER - and PR -/HER2 +	270 (7.54%)	226 (7.18%)	44 (10.14%)	
	Subgroup 4: ER – and PR -/HER2 –	632 (17.65%)	516 (16.40%)	116 (26.73%)	
Pathology	Invasive ductal carcinoma	3358 (93.80%)	2943 (93.55%)	415 (95.62%)	0.093
	Invasive lobular carcinoma	222 (6.20%)	203 (6.45%)	19 (4.38%)	
Lymph vascular invasion	No	2426 (67.77%)	2186 (69.49%)	240 (55.30%)	<0.001
	Yes	1154 (32.23%)	960 (30.51%)	194 (44.70%)	
Type of surgery	Breast conserving surgery	2548 (71.17%)	2358 (74.95%)	190 (43.78%)	<0.001
	Modified radical mastectomy	1032 (28.83%)	788 (25.05%)	244 (56.22%)	
Chemotherapy	None	472 (13.18%)	452 (14.37%)	20 (4.61%)	<0.001
	Adjuvant	2586 (72.23%)	2247 (71.42%)	339 (78.11%)	
	nonadjuvanted	522 (14.58%)	447 (14.21%)	75 (17.28%)	
Radiotherapy	None	137 (3.83%)	115 (3.66%)	22 (5.07%)	<0.001
	External	2937 (82.04%)	2533 (80.51%)	404 (93.09%)	
	Intraoperative	506 (14.13%)	498 (15.83%)	8 (1.84%)	
Hormonotherapy	No	714 (19.94%)	600 (19.07%)	114 (26.27%)	<0.001
	Yes	2866 (80.06%)	2546 (80.93%)	320 (73.73%)	

The frequency (percentage) was used to describe the categorical data. Numeric variables were presented using mean ± SD. The association between status (censored and deceased) and categorical variables was evaluated using Pearson Chi-Square test. The independent test was used to compare the mean of age between groups.

### 3.2 Prognosis feature importance

**Figure 2:**
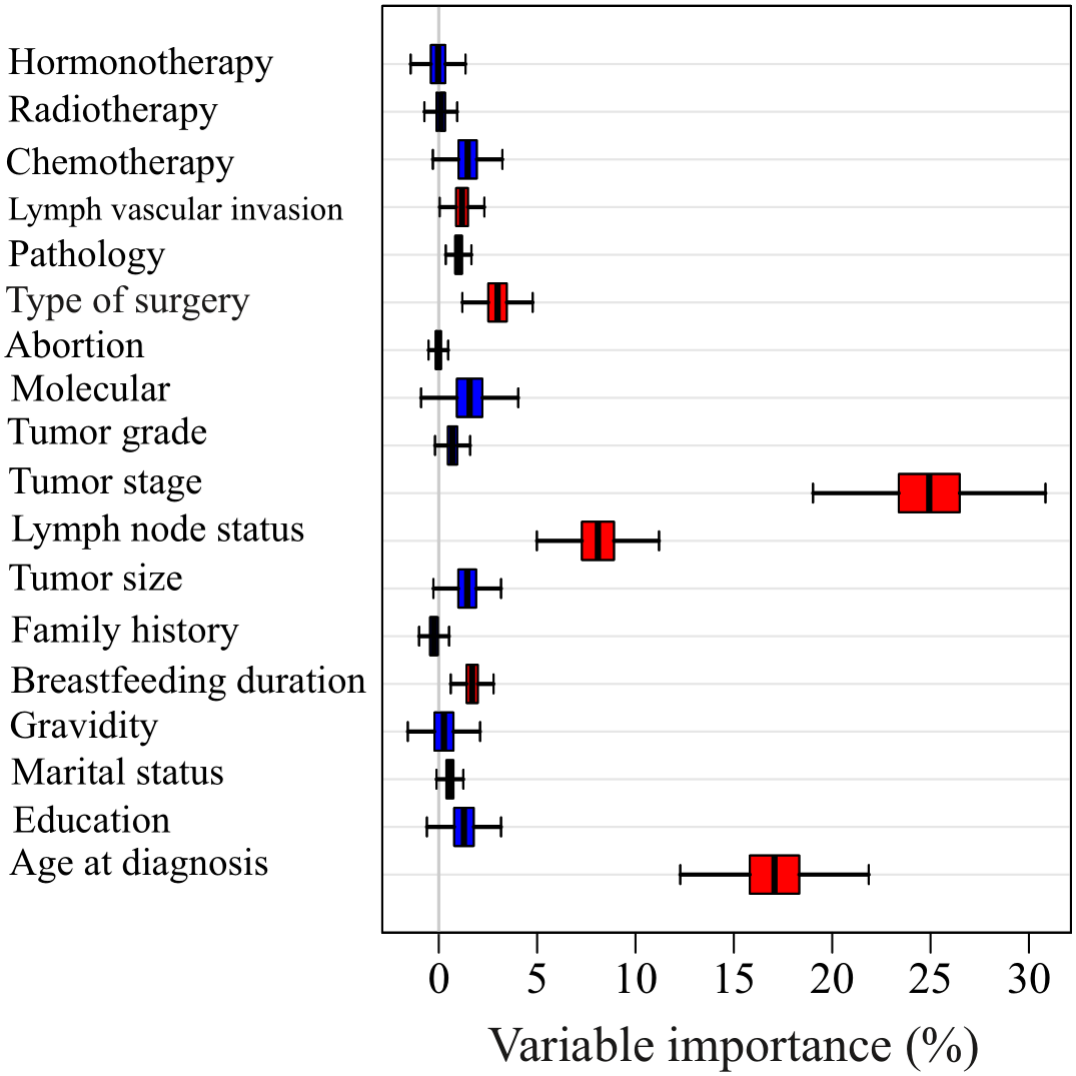
The Mean decrease accuracy of each variable in predicting the survival time using random forest method.

The most significant factors affecting survival probability were identified by selecting the variables with the highest mean decrease in accuracy, as shown in **Figure 2**. The results revealed that six variables, including lymph vascular invasion, type of surgery, tumor stage, lymph node status, breastfeeding duration, and age at diagnosis (plus molecular subtypes), had the highest mean decreases in accuracy and were therefore considered to be the most important factors. Moreover, tumor stage, age at diagnosis, and lymph node status (plus molecular subtypes) were identified as the top three important variables with larger mean decreases in accuracy compared to other variables. To categorize variables into different molecular subtypes, the molecular subtype status was taken into account to perform modelling along with the obtained important factors from the random forest algorithm.

### 3.3 The prediction performance using all variables

**Table 3** presents the outcomes of models’ performances that were developed using all variables. These results show the optimal C-index and IBS metrics of the trained models. According to the C-index, Nnet-survival and NMTLR models with a score of 0.77 had the highest scores; nevertheless, the Nnet-survival had the best overall performance in terms of IBS with a score of 0.14. Ultimately, the Nnet-survival model was selected as the best-performing model for predicting survival data using all variables.

**Table 3:** Performance of five models on Breast Cancer test set.

NO.	Model	All variables		Using 7 important variables		Using 4 important variables	
		C-index	IBS	C-index	IBS	C-index	IBS
1	DeepHit	0.72	0.18	0.76	0.18	0.75	0.20
2	N-MTLR	**0.77**	0.19	0.71	0.20	0.72	0.21
**3**	**Nnet-survival**	**0.77**	**0.14**	**0.76**	**0.18**	**0.76**	**0.16**
4	DeepSurve	0.75	0.15	0.74	0.20	0.74	0.19
5	Cox-Time	0.75	0.15	0.74	0.16	0.75	0.16

The bold values represent the C-index and IBS of the best-performing model with all independent variables, the six most important variables and the three most important variables in conjunction with molecular subtype status, as determined by feature importance analysis.

**Figure 3:**
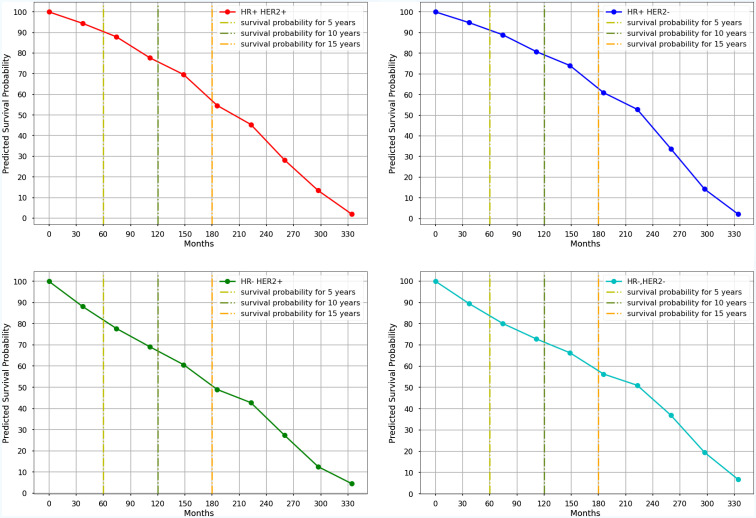
The Kaplan-Meyer curves of mean survival probabilities for each molecular subtype predicted by the Nnet-survival model, using all variables, during 30 years of follow-up (Red graph: Luminal B, Blue graph: Luminal A, Green graph: HER2-Enriched, Aqua graph: Triple-Negative).

**Figure 3** shows the Kaplan-Meier curves of mean survival probability in 10-time intervals for each molecular subtype predicted by the Nnet-survival model, which was developed by all variables. **Table 4** displays the 5-, 10-, and 15-year predicted survival probabilities for each molecular subtype.

**Table 4:** Mean survival probability of each molecular subtype in different time periods predicted by the Nnet-survival model developed with all timeindependentvariables.

NO.	Molecular Subtypes	5-years survival	10-years survival	15-years survival
1	luminal B	**91%**	77%	50%
2	luminal A	**91%**	**80%**	**60%**
3	HER2-enriched	82%	70%	54%
4	Triple negative	82%	72%	59%

The bold values represent the highest values of mean survival probabilities for each molecular subtype (luminal A, ER-positive/or PR-positive/HER2-negative; luminal B, ER-positive/or PR-positive/HER-positive; triple negative, ER-negative/PR-negative/HER-negative; HER2-enriched, ER negative/PR-negative/HER2-positive).

### 3.4 The prediction performance using important variables

**Figure 4:**
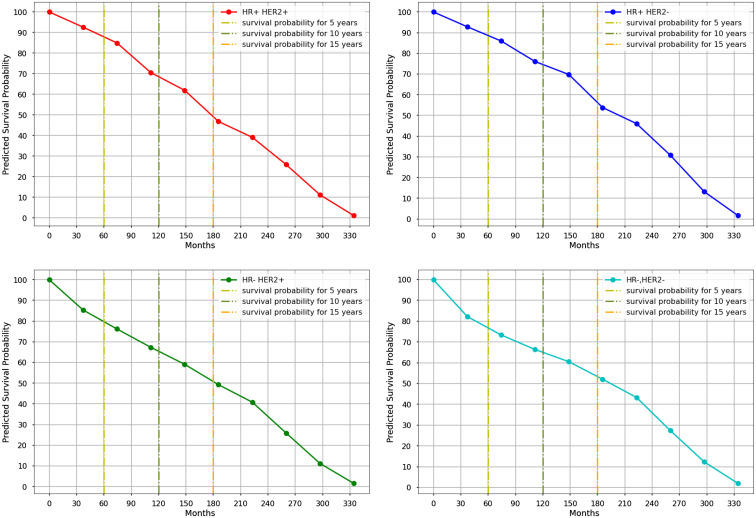
The Kaplan-Meyer curves of mean survival probabilities for each molecular subtype predicted by the Nnet-survival model, using three important variables, during 30 years of follow-up. (Red graph: Luminal B, Blue graph: Luminal A, Green graph: HER2-Enriched, Aqua graph: Triple-Negative).

With a C-index of 0.76 and an IBS of 0.18, the Nnet-survival model demonstrated the highest performance when utilizing seven significant variables in its development, while also achieving a C-index and IBS score of 0.76 and 0.18 respectively when using only the four primary variables (**Table 3**). **Figure 4** and **Table 5** display the 5-, 10- and 15-year mean survival probabilities predicted by the Nnet-survival model developed with the three most important variables along with molecular subtype status.

**Table 5:** Mean survival probability of each molecular subtype in different time periods predicted by Nnet-survival model developed with three most important variables.

NO.	Molecular Subtypes	5-years survival	10-years survival	15-years survival
1	luminal B	**91%**	76%	65%
2	luminal A	**91%**	**79%**	**69%**
3	HER2-enriched	88%	76%	68%
4	Triple negative	84%	74%	66%

The bold values represent the highest values of mean survival probabilities for each molecular subtype (luminal A, ER-positive/or PR-positive/HER2-negative; luminal B, ER-positive/or PR-positive/HER-positive; triple negative, ER-negative/PR-negative/HER-negative; HER2-enriched, ER negative/PR-negative/HER2-positive).

## 4 Discussion

This is the largest study so far in Iran, predicting survival probabilities of BC patients in four molecular subtypes, defined by ER, PR, and HER2 status. All models (DeepHit, NMTLR, Nnet-survival, DeepSurve and Cox-Time) yielded very close C-index and IBS, with Nnet-survival slightly higher using both total and the most important variables.

The purpose of this study was to use machine learning methods to predict the survival probability of BC patients. Previous studies have relied on traditional models, such as the Cox regression model (32), to analyze time-to-event data. However, the Cox model and other parametric survival distributions are limited in their linear and inflexible form. The linearity of the risk function in survival applications, such as treatment recommendations for patients with different conditions, may be very simplistic and does not provide an accurate estimate of survival prediction due to the presence of complex patterns and non-linear relationships between different variables (16).

Recent studies have employed machine learning techniques to develop classification models that predict survivability (33–36). These models have mainly focused on interpretability to improve accuracy, and the outcome measure is often evaluated at a single time point. Furthermore, dealing with censored data in survival models based on machine learning classification models is frequently not discussed, and the flexibility associated with modeling the event probabilities as a function of time have been mostly neglected (34, 37–44). Additionally, previous studies on BC survival prediction did not consider some important issues such as feature importance, competent pre-processing steps, and using adequate sample size when developing their models (45, 46). In contrast, this study conducted comprehensive data pre-processing and variable selection procedure to develop an optimal and reliable model. Moreover, a grid search method was used to tune the hyperparameters of the neural network.

In this study, we utilized Random Forest feature importance to identify the most influential variables on survival time. Random Forest has been used for feature importance applications in many BC surveillance studies (47–50), due to its capacity to effectively process highly non-linear data (51, 52). We found that the six most important variables were age at diagnosis, tumor stage, axillary lymph node metastasis, type of surgery, lymph vascular invasion, and breast-feeding duration. These same variables were also defined as the most important variables in previous studies related to BC (35, 53). Furthermore, our findings suggested that the top three critical variables affecting survival probabilities were age at diagnosis, stage, and axillary lymph node metastasis. This is consistent with other studies which have demonstrated stage and axillary lymph node metastasis as the most significant predictors of BC prognosis (54, 55).

Subsequent studies have indicated that the chance of survival for BC patients decreased with increasing age at the diagnosis (56). Previous studies have demonstrated that age is an important risk factor in BC prognosis (57–60).However, due to the lack of consensus on different thresholds for age and the use of broad age groups, the role of this predictor remains controversial. Some studies have focused only on young or older women rather than all age groups (61, 62). In this study, we avoided categorizing the patients’ age and included all age groups. Additionally, tumor stage is also an important prognostic factor affecting BC survival time (63, 64). A study conducted in the Netherlands, found that tumor stage can affect overall survival in the current era of effective systemic therapy (65). Furthermore, metastasis to axillary lymph nodes is another chief factor affecting BC prognosis (66, 67). It has been noted that nearly 8% to 30% newly diagnosed BC are at an advanced stage, with extensive axillary lymph node metastasis (68). Results from a study in China found that BC patients with lower lymph node metastasis had more prolonged overall survival, disease-free survival, and distant metastasis-free survival compared to patients with more involvement of lymph node metastasis (69).

The models generated very close C-index and IBS scores trained with all 18, seven, and four variables. Furthermore, the survival probabilities of molecular subtypes acquired from the Nnet-survival model trained with the three most important variables showed similar patterns to results from the model trained with all variables (**Supplementary Figure S1**, **S2**). This suggests that using only the three most important variables instead of a large number of variables could result in a more robust and accurate model with less complexity.

Our results showed that the luminal A subtype had the highest predicted survival probabilities across the four molecular subtypes, with 91%, 80%, and 60% in 5-, 10-, and 15-year follow-ups, respectively. Our results (**Supplementary Figure S1**) also showed that the luminal B subtype followed a similar trend as luminal A for the first five years, after which the predicted survival probability decreased steadily and reached 77% and 50% in 10- and 15 years, respectively. This is consistent with Christine Inwald et al.’s study, which found that overall survival rates of luminal B (80.3%) and luminal A (87.5%) subtypes declined over 7-year where luminal A showed the best overall survival (70). Additionally, we found that the survival probabilities for triple-negative and HER2-enriched subtypes had a similar pattern with 82% in 5-year follow-up. The decreasing trend continued in both HER2-enriched and triple-negative subtypes; however, it was more pronounced in the HER2-enriched subtype with 70% and 54% predicted survival probabilities in 10- and 15-year follow-up, respectively. These findings confirm previous studies’ findings which indicated that the HER2-enriched subtype had a worse prognosis than the Luminal A subtype, although they were based on much shorter follow-up times (10, 71, 72). Moreover, the triple-negative subtype had a slight decrease with 72% predicted survival probability in 10-year and had the least decline compared to other subtypes between 10 and 15 years of follow-up. We observed higher survival probabilities in patients with luminal A and triple-negative subtypes after 12 years of follow-up, which suggests that successful therapeutic management is possible when considering all prognostic factors. Other related studies have reported that the mortality rate for the triple-negative subtype is initially high but gradually decreases over time, while the mortality rate for the luminal A subtype remains almost constant (8, 9).

Many studies have indicated that HER2-positive subtypes, regardless of ER and PR status, are associated with a poorer prognosis than other subtypes (7, 73, 74). Our findings showed that HER2 is a time-relevant factor and the survival probabilities for HER2-positive subtypes depend on both the time and ER/PR status. It is possible that hormonal therapy (tamoxifen or aromatase inhibitor) may have improved survival probabilities for the luminal B subtype in the first 12 years of follow-up. Additionally, it is conceivable that the earlier deaths of HER2-positive patients could be attributed to the unavailability of Trastuzumab between 1991 to 2009; this drug was approved by the US FDA in 2005 and became available in Iran in 2009 (75). Despite the advantages of Trastuzumab and other anti-HER2 therapies, a reduction in survival probabilities for luminal B subtype compared with HER2-enriched after 12 years suggests that these treatments, along with hormonal therapy, may not be reliable treatment strategies for luminal B patients. Therefore, it is recommended that HER2 status should be taken into greater consideration during BC treatment periods. For example, the cases whose life expectancy is restricted to less than five or ten years should not be evaluated by HER2 and its common treatments.

The current study was limited by potential biases of registry-based retrospective analyses (76). The major limitation, like any long-term retrospective analyses, was data censorship, which was addressed by using time-to-event survival models to model the relationships between covariates and individual survival time distributions. Furthermore, due to the varying coring and staining methods used in laboratories conducting IHC tests, some misclassification of cancer subtypes is unavoidable. Moreover, the relatively small number of patients in late time intervals posed a limitation to this study. As HER2 oncogene is positive in about 20% of primary BCs (74), the number of patients with HER2-positive subtypes decreased over time due to the decrease in total amount of data. Additionally, the number of events in the dataset decreased dramatically after 15 years of (**Supplementary Figure S3**), making it difficult to train a reliable survival model for data in late intervals (more than 15 years of follow-up). Despite all limitations, this study was able to assess BC patient survival over a long-term follow-up period and reveal differences across BC subtypes with greater precision.

## 5 Conclusions

In conclusion, we developed time-to-event deep learning models using data from a large institutional BC dataset in Iran to evaluate the survival prediction models. The best-performing model was used to predict survival probability in four BC molecular subtypes, in order to compare survival patterns over different time intervals since diagnosis. Our findings provide healthcare providers with the ability to determine patients’ survivability, better understand the effect of each treatment on different molecular subtypes and prevent unnecessary interventions for high-risk, particularly those with HER2 positive status, based on their molecular receptor status.

## 6 Recommendations for future studies

More broadly, our research is also needed to determine the survival probabilities for each subtype in different age and stage categories, in order to enable clinicians to make individualized treatment decisions that could influence clinical outcomes in patients’ short- and long-term survival. Moreover, the data used in this study was collected from an academic center in Iran’s capital, and therefore does not reflect the entire Iranian population. Considerably, further research will need to be conducted to compare outcome between patients with different backgrounds in Iran. However, the ultimate goal is to focus on other Asian countries where such research has barely been carried out. Additionally, existing survival models should be translated into new prediction tools for healthcare organizations, such as PREDICT (77), which enables the incorporation of BC molecular status into predictions of BC survival. Furthermore, more trials should be conducted to estimate the benefits and risks of hormonal therapy, anti-HER2 therapy, and chemotherapy for patients with different molecular profiles.

## Data availability statement

The raw data supporting the conclusions of this article will be made available by the authors, without undue reservation. The python (v3.6) source codes used to develop time-to-event models are deposited in GitHub (https://github.com/sabazarean/Breast-Cancer-Survival-Prediction).

## Ethics statement

Written informed consent was obtained from the individual(s) for the publication of any potentially identifiable images or data included in this article.

## Author contributions

MEA and SZ conceived and planned the experiments. SZ, MAL, PM and MA carried out the analyses and experiments. SZ and AA contributed to the collection of the samples and clinical data. SZ, MA, and MEA contributed to the interpretation of the results. SZ, and MAL and PM took the lead in writing the manuscript. MEA and HE revised the manuscript. MEA and FA supervised the project. All authors reviewed and confirmed the manuscript.

## Footnotes

1
https://github.com/havakv/pycox

2
https://github.com/scikit-learn/scikit-learn

## Supplementary material

Supplementary Figure 1 The comparison of Kaplan-Meyer curves of mean survival probabilities for each molecular subtype predicted by the Nnet-survival model, using all variables, during 30 years of follow-up. (Red graph: Luminal B, Blue graph: Luminal A, Green graph: HER2-Enriched, Aqua graph: Triple-Negative)

Supplementary Figure 2 The comparison of Kaplan-Meyer curves of mean survival probabilities for each molecular subtype predicted by the Nnet-survival model, using the three important variables, during 30 years of follow-up. (Red graph: Luminal B, Blue graph: Luminal A, Green graph: HER2-Enriched, Aqua graph: Triple-Negative)

Supplementary Figure 3 The distribution of the number of patients for each molecular subtype during the 30 years of follow-up.
