# Supplementary figures and images for: Time-related survival prediction in molecular subtypes of breast cancer using time-to-event deep-learning-based models
Source: Front Oncol. 2023 Jun 5;13:1147604. doi: 10.3389/fonc.2023.1147604 (PMC10277681; doi:10.3389/fonc.2023.1147604)

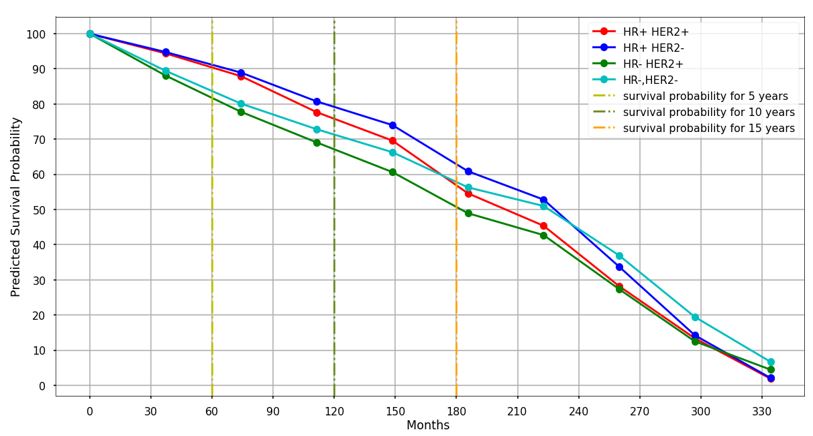

Supplement: Supplementary Figure 1 — The comparison of Kaplan-Meyer curves of mean survival probabilities for each molecular subtype predicted by the Nnet-survival model, using all variables, during 30 years of follow-up. (Red graph: Luminal B, Blue graph: Luminal A, Green graph: HER2-Enriched, Aqua graph: Triple-Negative) [file Image_1.jpeg]

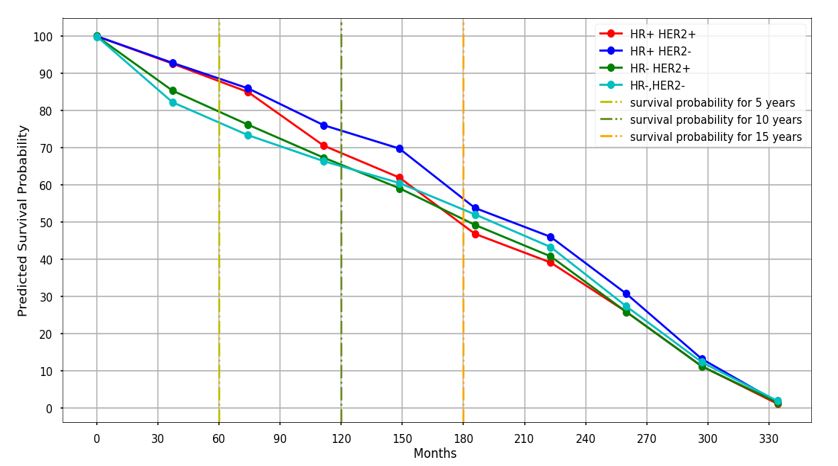

Supplement: Supplementary Figure 2 — The comparison of Kaplan-Meyer curves of mean survival probabilities for each molecular subtype predicted by the Nnet-survival model, using the three important variables, during 30 years of follow-up. (Red graph: Luminal B, Blue graph: Luminal A, Green graph: HER2-Enriched, Aqua graph: Triple-Negative) [file Image_2.jpeg]

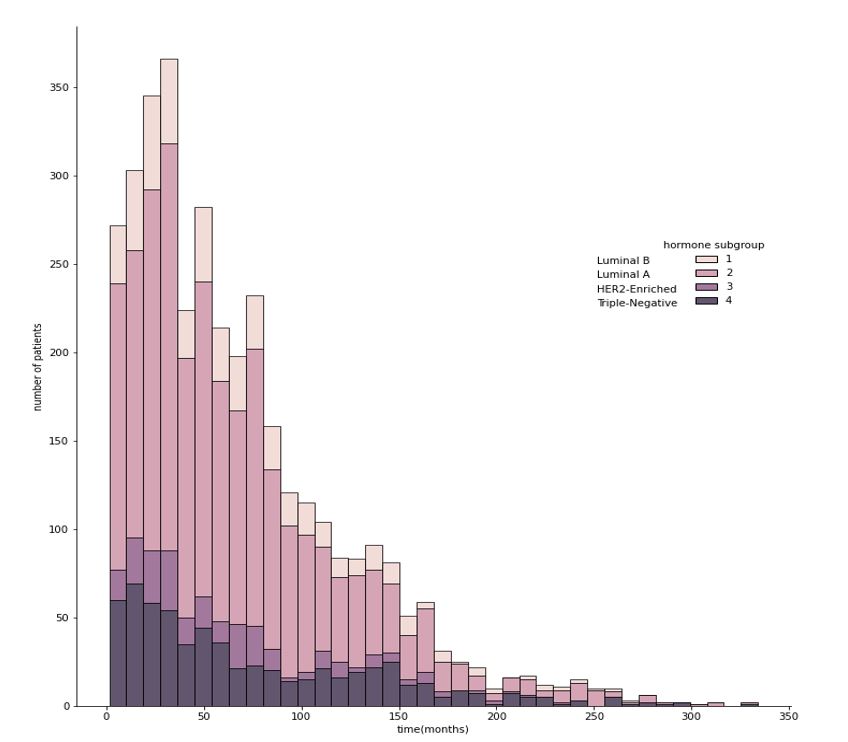

Supplement: Supplementary Figure 3 — The distribution of the number of patients for each molecular subtype during the 30 years of follow-up. [file Image_3.jpeg]
